# Examining the clinical relevance of metformin as an antioxidant intervention

**DOI:** 10.3389/fphar.2024.1330797

**Published:** 2024-02-01

**Authors:** Angelika Buczyńska, Iwona Sidorkiewicz, Adam Jacek Krętowski, Agnieszka Adamska

**Affiliations:** ^1^ Clinical Research Centre, Medical University of Bialystok, Bialystok, Poland; ^2^ Clinical Research Support Centre, Medical University of Bialystok, Bialystok, Poland; ^3^ Department of Endocrinology, Diabetology and Internal Medicine, Medical University of Bialystok, Bialystok, Poland

**Keywords:** metformin, oxidative stress, antioxidant intervention, oxidative status, novel treatment

## Abstract

In physiological concentrations, reactive oxygen species play a vital role in regulating cell signaling and gene expression. Nevertheless, oxidative stress is implicated in the pathogenesis of numerous diseases and can inflict damage on diverse cell types and tissues. Thus, understanding the factors that mitigate the deleterious effects of oxidative stress is imperative for identifying new therapeutic targets. In light of the absence of direct treatment recommendations for reducing oxidative stress, there is a continuing need for fundamental research that utilizes innovative therapeutic approaches. Metformin, known for its multifaceted beneficial properties, is acknowledged for its ability to counteract the adverse effects of increased oxidative stress at both molecular and cellular levels. In this review, we delve into recent insights regarding metformin’s antioxidant attributes, aiming to expand its clinical applicability. Our review proposes that metformin holds promise as a potential adjunctive therapy for various diseases, given its modulation of oxidative stress characteristics and regulation of diverse metabolic pathways. These pathways include lipid metabolism, hormone synthesis, and immunological responses, all of which may experience dysregulation in disease states, contributing to increased oxidative stress. Furthermore, our review introduces potential novel metformin-based interventions that may merit consideration in future research. Nevertheless, the necessity for clinical trials involving this drug remains imperative, as they are essential for establishing therapeutic dosages and addressing challenges associated with dose-dependent effects.

## 1 Introduction

The hypoglycemic activity of metformin (a synthetic derivative of guanidine) was first described in 1968. Since its approval by the U.S. Food and Drug Administration (FDA) in 1994, metformin has become a first-line treatment for type 2 diabetes mellitus (T2DM) (Clarke and Duncan, 1968; Corcoran and Jacobs, 2021). Nowadays, metformin has also been approved for several non-FDA-approved indications, including gestational diabetes, management of antipsychotic-induced weight gain, and treatment of polycystic ovary syndrome (PCOS) (Dutta et al., 2001; Sirmans and Pate, 2014; Corcoran and Jacobs, 2021). Beyond its well-established antihyperglycemic impact, metformin demonstrates anti-inflammatory and antioxidant activities, contributing to its potential benefits in conditions associated with inflammation and oxidative stress (Bharath and Nikolajczyk, 2021). Notably, metformin is linked to cardioprotective effects, modest weight loss, and potential anticancer properties (Mallik and Chowdhury, 2018). It is also being explored for neuroprotective effects and its role in promoting healthy aging (Foretz et al., 2023). Additionally, metformin may influence gut microbiota, further implicating its broad impact on various physiological processes (Triggle et al., 2022). While these pleiotropic effects hold promise for additional therapeutic use of metformin, ongoing research is crucial to fully understand the underlying mechanisms and determine their clinical significance in diverse medical conditions (Anedda et al., 2008; Diniz Vilela et al., 2016; Forman and Zhang, 2021). Recent studies involving newly diagnosed T2DM patients subjected to a 12-month observation period during metformin intervention have elucidated that metformin usage proves more efficacious in attenuating oxidative stress when compared to lifestyle modification alone. Additionally, within this observational timeframe, individuals undergoing metformin intervention manifested notable reductions in concentrations of advanced oxidation protein products (AOPP) and advanced glycation end products (AGE) in comparison to the control group (Mirmiranpour et al., 2013). According to study conducted by Adeshara et al., augmented antioxidant properties were evident following a 3-month period of metformin administration in patients with T2DM patients (Adeshara et al., 2020). Given the existing inconsistencies in the results of contemporary studies, a thorough review of fundamental scientific investigations is imperative to assess the antioxidant properties of metformin accurately. The current lack of consensus in findings underscores the necessity for a meticulous examination using basic science studies. This review aims to synthesize and analyze the diverse outcomes reported in these studies, thereby providing a comprehensive perspective on the potential antioxidative effects of metformin. Through a detailed scrutiny of underlying molecular mechanisms and variations in experimental methodologies, this review aspires to contribute substantively to the elucidation of metformin’s role as an antioxidant.

## 2 Materials and methods

The literature review was conducted using various databases including Scopus, Web of Science, PubMed, Directory of Open Access Journals (DOAJ), ClinicalTrials.gov, and Science Direct. The literature search was conducted using relevant keywords such as “metformin intervention," “oxidative stress," “antioxidant capacity," and “antioxidation." Exclusion criteria for the review included incorrect formulated conclusions, inaccurate study group plurality, unclear criteria for patient inclusion/exclusion, and inconsistent research methodology. The review protocol was registered in the PROSPERO database (CRD42022299568). Thus, all PROSPERO guidelines were incorporated during the execution of the literature review (Schiavo, 2019).

## 3 Multifaceted effects of metformin: understanding its pleiotropic properties

Metformin provides hypoglycemic effect through various mechanisms, such as inhibition of gluconeogenesis in the liver, increased glucose uptake in skeletal muscle and hepatocytes, decreased glucose absorption in the gastrointestinal tract (Viollet et al., 2012; McCreight et al., 2016; Lv and Guo, 2020). The metformin intervention also plays a role in glycerol-3-phosphate dehydrogenase and selectively inhibits the substrate (glycerol and lactate) involved in hepatic gluconeogenesis (Lamoia and Shulman, 2021). Its intervention is also associated with the transcriptional regulation of hepatic gluconeogenesis through antagonization of hepatic glucagon signaling. This mechanism involves reducing cyclic adenosine monophosphate (AMP) accumulation and preventing CREB-mediated transcription of gluconeogenic genes, leading to reduced glucose 6-phosphatase (G6pc) and phosphoenolpyruvate carboxykinase 1 (Pck1) expression (He et al., 2009; Miller et al., 2013; Johanns et al., 2016). Metformin indirectly regulates hepatic gluconeogenesis by controlling glycerol delivery to the liver through white adipose tissue (WAT) lipolysis. In this process, glycerol is phosphorylated and converted to dihydroxyacetone phosphate (DHAP) by mitochondrial glycerol-3-phosphate dehydrogenase (GPD2) in a redox-dependent manner. Studies on rodents by Madiraju et al. showed that metformin treatment increased plasma glycerol and decreased hepatic glycerol-3-phosphate (G3P) activity, indicating reduced gluconeogenesis (Madiraju et al., 2014; Madiraju et al., 2018). Furthermore, metformin enhances this process by promoting an increase in the secretion of glucagon-like peptide 1 (GLP-1). GLP-1 is a hormone that plays a crucial role in regulating blood glucose levels and insulin secretion. By augmenting GLP-1 secretion, metformin contributes to the improvement of glucose metabolism and insulin sensitivity, which are vital factors in managing conditions such as T2DM. This combined effect of metformin on glycerol-3-phosphate dehydrogenase, substrate inhibition, and GLP-1 secretion makes it an essential therapeutic option for controlling hepatic gluconeogenesis and maintaining glucose homeostasis in individuals with insulin resistance or T2DM (Bahne et al., 2018). In addition to its other beneficial effects, metformin also enhances tissue sensitivity to insulin through an increase in the activity of the insulin receptor tyrosine kinase. The insulin receptor is a transmembrane protein found on the surface of target cells, and its activation is a critical step in insulin signaling. When insulin binds to its receptor, it triggers a series of molecular events, leading to the activation of the insulin receptor tyrosine kinase. This kinase then adds phosphate groups to specific tyrosine residues on the insulin receptor and other intracellular substrates, initiating a cascade of signaling pathways that promote glucose uptake, glycogen synthesis, and other insulin-dependent cellular responses (He et al., 2009). Metformin’s ability to boost the activity of insulin receptor tyrosine kinase enhances the responsiveness of cells to insulin’s actions. As a result, tissues become more receptive to glucose uptake and utilization, which is particularly beneficial in individuals with insulin resistance or T2DM. By improving insulin sensitivity and glucose utilization in tissues, metformin plays a crucial role in managing blood glucose levels (Mulherin et al., 2011).

Apart from its hypoglycemic effects, metformin has been shown to have several other beneficial effects on lipid profile, immune and coagulation responses, and hormone synthesis (Akinci et al., 2008; Buczyńska et al., 2022). This is due to the activation of AMPK, which regulates many metabolic pathways (Musi et al., 2002; Vancura et al., 2018; Meyer et al., 2020). Sterol regulatory element binding proteins (SREBP), which regulates the expression of genes involved in lipogenesis, is also positively regulated by insulin and negatively regulated by AMPK activity in the intestine (Musi et al., 2002). Metformin has been shown to decrease the synthesis of intestine-derived triglycerides-rich lipoproteins, which leads to a decrease in plasma concentrations of chylomicrons and chylomicron remnant lipoprotein fractions (Zhang et al., 2018). This is achieved by a decrease in the expression of SREBP-1c, SREBP-2, ACC1, and Apo A-IV (van Stee et al., 2018; Vancura et al., 2018; Zhang et al., 2018; Jia et al., 2019; Buczyńska et al., 2020; Meyer et al., 2020). Metformin also enhances the fluidity of cell membranes (Marc et al., 2014) and inhibits the inflammatory process by increasing plasma fibrinolytic activity, reducing the concentration of plasminogen activator inhibitor-1 (PAI-1), preventing excessive activation of platelets, and reducing endothelial damage (Hirsch et al., 2013; Bai and Chen, 2021), and these properties were proven during clinical trials among T2DM and metabolic syndrome patients.

Metformin also has a significant impact on the PCOS, decreasing luteinizing hormone and androgen synthesis, resulting in normalizing the menstruation cycle (Vause et al., 2010; Shao et al., 2014). Metformin treatment was associated with a decrease in the levels of serum thyroid-stimulating hormone (TSH) in diabetic patients possibly by enhancing the effects of thyroid hormones in the pituitary and activating the adenosine monophosphate-activated protein kinase (AMPK) (Meng et al., 2017). Furthermore, metformin treatment has been found to be associated with a decrease in cell proliferation and a reduction in the secretion of growth hormone (GH) (An et al., 2017). Additionally, metformin has been shown to regulate the pulsatile release of cortisol and allopregnanolone (Genazzani et al., 2009). These effects are of significant interest as dysregulation of cell proliferation, GH secretion, and cortisol levels can have implications for various health conditions, including metabolic disorders and hormonal imbalances.

**FIGURE 1:**
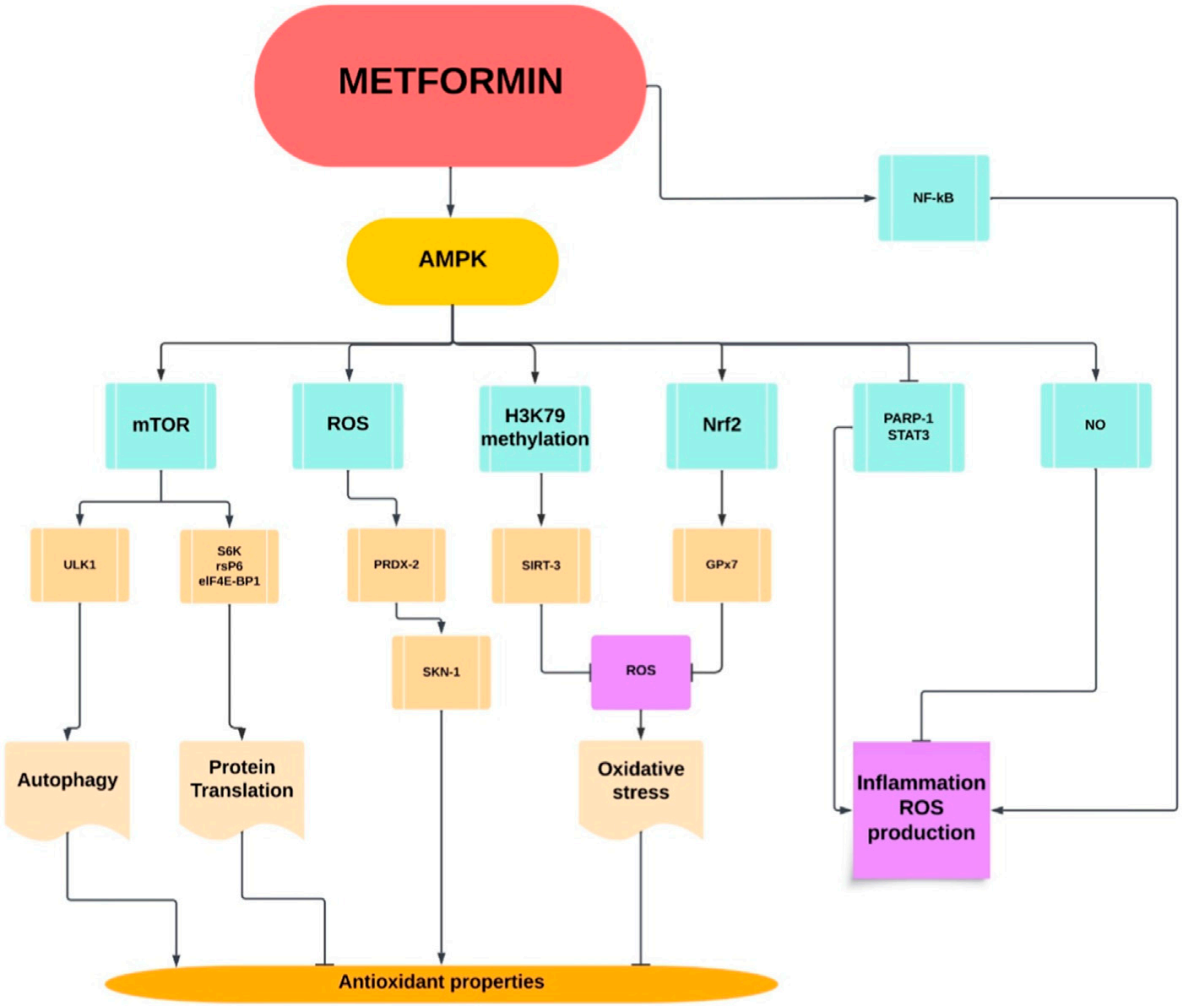
Pleiotropic metformin properties.

The pleiotropic effects of metformin are achieved through its ability to modulate multiple factors at the molecular level. AMPK serves as a central regulator, influencing downstream targets such as eukaryotic translation initiation factor 4E-binding protein 1 (eIF-4E-BP1), glutathione peroxidase 7 (GPx7), mechanistic target of rapamycin (mTOR), nuclear factor erythroid 2-related factor 2 (Nrf2), and nuclear factor-kappa B (NF-kB). AMPK activation by metformin impacts diverse cellular processes, including translation initiation, antioxidant defense, nutrient sensing, and inflammatory responses. Additionally, metformin’s influence on nitric oxide (NO) production, poly (ADP-ribose) polymerase 1 (PARP-1) inhibition, and modulation of reactive oxygen species (ROS) and sulfur species further contributes to its multifaceted actions. The integration of these molecular mechanisms, orchestrated by AMPK, underscores metformin’s comprehensive therapeutic benefits beyond glycemic control, encompassing anti-inflammatory, antioxidant, and cytoprotective effects (Figure 1).

AMPK: AMP-activated protein kinase; elif-4E-BP1: Eukaryotic translation initiation factor 4E-binding protein 1; GPx7: Glutathione peroxidase 7; mTOR: Mechanistic target of rapamycin; Nrf2: Nuclear factor erythroid 2-related factor 2; NK-kB: Nuclear factor-kappa B; NO: Nitric oxide; PARP-1: Poly (ADP-ribose) polymerase 1; PRDX-2: Peroxiredoxin-2; ROS: Reactive oxygen species; rsPG: Reactive sulfur species; SGK: Serum and glucocorticoid-regulated kinase; SIRT3: Sirtuin 3; SKN-1: Skinhead-1; STAT3: Signal transducer and activator of transcription 3; ULK1: Unc-51 like autophagy activating kinase 1.

While metformin has been demonstrated to improve mitochondrial respiration, it is worth noting that higher doses of metformin have been associated with a contrary effect, namely, reduced mitochondrial respiration. This reduction in mitochondrial respiration is attributed to metformin’s ability to decrease adenine nucleotide levels. Mitochondria are essential cellular organelles responsible for generating energy in the form of adenosine triphosphate (ATP) through oxidative phosphorylation. Metformin has been recognized for its positive impact on mitochondrial function by enhancing respiratory chain activity and promoting efficient ATP production, which is beneficial for cellular energy metabolism. However, at higher doses, metformin has been found to interfere with the levels of adenine nucleotides, including adenosine diphosphate (ADP) and ATP. Lower ADP levels can inhibit mitochondrial respiration and subsequently lead to a reduction in ATP synthesis. The dose-dependent effects of metformin on mitochondrial function underscore the importance of careful dosage management in clinical settings (Luengo et al., 2014). Additionally, metformin inhibits complex I-dependent respiration and mitochondrial glycerophosphate dehydrogenase (mGPDH) and activates sirtuin 1 (SIRT1) and sirtuin 3 (SIRT3), making it directly involved in oxidative stress modulation (Andrzejewski et al., 2014). The mechanism by which metformin may increase oxidative stress in certain circumstances involves several factors. While metformin is generally known for its beneficial effects in reducing oxidative stress and improving cellular metabolism, there are specific situations where it appears to have the opposite effect. In white adipose tissue collected from patients with T2DM or in certain experimental conditions using H4II rat hepatocellular carcinoma, metformin has been observed to induce oxidative stress. This paradoxical effect could be attributed to mitochondrial dysfunction, complex cellular interactions (the balance between antioxidant defenses and ROS generation might shift towards increased oxidative stress due to specific metabolic or signaling alterations induced by metformin), cellular adaptation (the response to metformin might differ depending on their metabolic state and the presence of pre-existing conditions) or genetic variability (individual genetic variations can influence metformin’s effects on oxidative stress) (Anedda et al., 2008).

Furthermore, it is important to note that the effectiveness of metformin varies depending on the specific tissue and the dosage used (Kefas et al., 2004; Klein et al., 2004). Different tissues may respond differently to metformin treatment, and the optimal dosage may vary for different individuals or medical conditions. Understanding the tissue-specific effects and the appropriate dosing regimens is crucial for maximizing the therapeutic benefits of metformin while minimizing potential adverse effects. Metformin has an oral bioavailability of approximately 50%, and after intestinal absorption, it accumulates in the liver (Ouslimani et al., 2005). As metformin continues to be a widely prescribed medication for conditions such as T2DM, PCOS, and metabolic syndrome, further research is warranted to explore its tissue-specific actions and refine the dosing strategies. Such insights will help optimize the use of metformin as a therapeutic intervention for various health conditions and pave the way for personalized treatment approaches in the future. Therefore, further research is still needed to determine the dose and dose-dependent modulation of oxidative stress by metformin. It is possible that metformin may increase antioxidant protection at low and medium doses, while high doses may intensify oxidative stress.

## 4 Unraveling the mechanisms behind metformin's antioxidant properties

**FIGURE 2:**
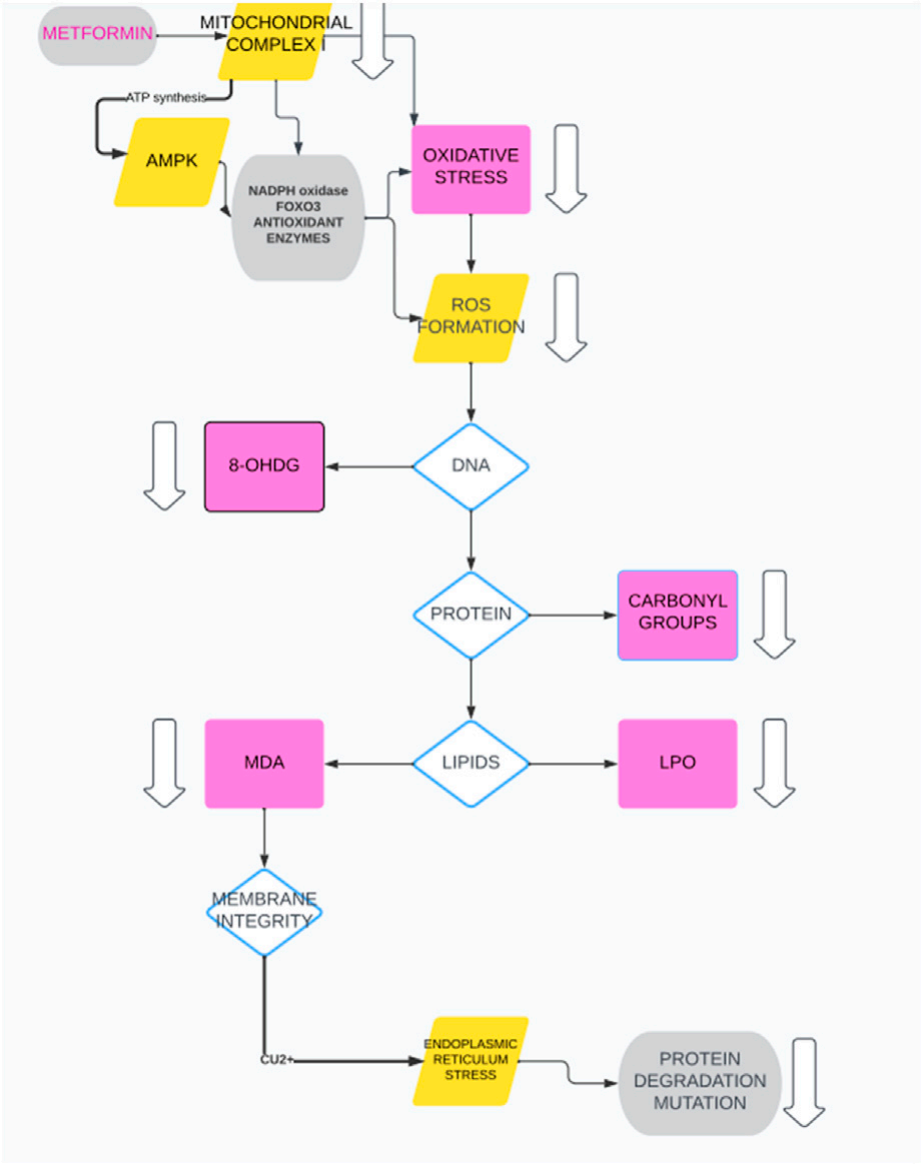
Metformin antioxidant properties.

Metformin can modulate oxidative stress by inhibiting ROS production through pleiotropic actions, including AMPK modulation, mitochondrial complex I inhibition, and increased antioxidant enzyme activity. This may limit the degradation of biological components, such as proteins, DNA and lipids, crucial for membrane integrity, as indicated by markers such as 8-Hydroxy-2′-deoxyguanosine (8-OHDG), AMPK activation, and modulation of several other key factors. Its influence on 8-OHDG suggests a potential in reducing oxidative DNA damage, showcasing a protective role against reactive oxygen species (ROS)-induced harm. The primary mechanism of metformin involves the activation of AMPK, enhancing cellular energy homeostasis and metabolic function. Additionally, interactions with copper ions (Cu^2+^) and modulation of transcription factor FOXO3 contribute to its antioxidant properties. Metformin mitigates lipid peroxidation, as evidenced by reduced levels of malondialdehyde (MDA), highlighting protection against oxidative stress-induced lipid damage. Through its impact on NADPH metabolism and reduction of ROS production, metformin plays a crucial role in maintaining cellular redox balance and defending against oxidative stress. Overall, metformin’s properties extend beyond glycemic control, encompassing antioxidative and cellular protective effects that contribute to its therapeutic benefits (Figure 2) (Samsuri et al., 2017; Bharath and Nikolajczyk, 2021; Liu et al., 2021; Buczyńska et al., 2022; Triggle et al., 2022; Foretz et al., 2023).

8-OHDG: 8-Hydroxy-2′-deoxyguanosine; AMPK: 5′AMP-activated protein kinase; ATP: Adenosine Triphosphate; Cu2+: Copper (II) ion; FOXO3; Forkhead box O3; LPO: Lipid Peroxidation; MDA: Malondialdehyde; NADPH: Nicotinamide Adenine Dinucleotide Phosphate; ROS: Reactive Oxygen Species.

### 4.1 *In vitro* experiments

In a study conducted by An et al., using human umbilical vein endothelial cells, it was found that metformin’s antioxidant properties, at a dose of 2 mmol/L, were achieved through specific mechanisms. The results indicated that metformin activated guanosine 5′-triphosphate cyclohydrolase 1 (GTPCH1), leading to the recoupling of endothelial nitric oxide synthase (eNOS). This recoupling of eNOS resulted in increased production of nitric oxide, which possesses antioxidant properties. Moreover, metformin inhibited nicotinamide adenine dinucleotide phosphate (NADPH) oxidase via an AMP-activated protein kinase (AMPK)-dependent pathway. NADPH oxidase is a key enzyme responsible for producing reactive oxygen species (ROS) that contribute to oxidative stress. By inhibiting NADPH oxidase, metformin helps to reduce the generation of harmful ROS, further contributing to its antioxidant effects (An et al., 2016). In a study conducted by Hou et al., the effect of metformin intervention (dose: 250 µM) on reducing reactive oxygen species (ROS) concentration in human aortic endothelial cells was investigated. The decrease in ROS levels was attributed to an increase in the concentration of the antioxidant thioredoxin (Trx). Furthermore, Trx was found to upregulate the expression of the forkhead transcription factor 3 (FOXO3) through the AMPK pathway. FOXO3 is a transcription factor that plays a role in regulating genes involved in cellular stress response and antioxidant defense. These results suggest that metformin exerts its antioxidant effects through the modulation of Trx and the subsequent activation of the AMPK-FOXO3 pathway, providing a mechanistic understanding of metformin’s antioxidative properties in human endothelial cells (Hou et al., 2010). On the contrary, Cheng et al. demonstrated that the reduction in oxidative stress induced by metformin (dose: 1 mM) is associated with the downregulation of NADPH Oxidase 4 (NOX4) expression. NOX4 is an enzyme responsible for producing reactive oxygen species (ROS) that contribute to oxidative stress in cells. The downregulation of NOX4 by metformin helps to decrease the generation of harmful ROS, providing an alternative mechanism for metformin’s antioxidative effects. These findings offer valuable insights into the diverse ways in which metformin can modulate oxidative stress in different cellular contexts (Cheng and Lanza-Jacoby, 2015). Furthermore, in a study by Ouslimani et al., where aortic endothelial cells were subjected to metformin intervention (dose: 1–5 μmol/L), it was observed that this treatment led to a reduction in intracellular reactive oxygen species (ROS) production. This effect was achieved through the regulation of NADPH oxidase activity. NADPH oxidase is an enzyme responsible for generating ROS, and metformin’s ability to modulate its activity contributed to the decrease in ROS levels, highlighting another mechanism by which metformin exerts its antioxidative properties in aortic endothelial cells (Ouslimani et al., 2005). In a study conducted by Bałdak et al., peripheral blood mononuclear human cells obtained from 10 healthy individuals were cultured with metformin (dose: 2 µM) for 24 h. The results showed a decrease in the expression of NADPH oxidase, an enzyme responsible for generating reactive oxygen species, indicating a reduction in oxidative stress. Furthermore, the metformin treatment led to an increase in the expression of various antioxidative enzymes, including superoxide dismutase (SOD), catalase (CAT), and glutathione peroxidase (GPx). These enzymes play crucial roles in neutralizing ROS and protecting cells from oxidative damage. The study suggests that metformin’s antioxidative effects in human blood cells involve downregulating NADPH oxidase and upregulating key antioxidative enzymes, which may contribute to its potential therapeutic benefits in managing oxidative stress-related conditions (Bułdak et al., 2016).

From the other hand, Anedda et al. examined metformin’s impact on white adipocytes and its influence on uncoupling protein 2 (UCP2) levels. The UCPs are transporters that decrease the efficiency of mitochondrial oxidative phosphorylation. UCP2 is believed to provide protection against oxidative stress, although alternatively, it may function in dissipating energy. Cultured rat adipocytes treated with metformin (dose: 4 mM) showed increased UCP2 and ROS levels, and decreased aconitase activity. Variations in UCP2 levels parallel ROS changes. Metformin increases lipolysis in cells, but only post-reduction in ROS and UCP2 levels. UCP2 appears unnecessary for fatty acid oxidation facilitation. Metformin treatment elevates UCP2 in white adipose tissue. In summary, metformin induces UCP2 overexpression in adipocytes, potentially minimizing oxidative stress due to drug-induced respiratory inhibition (Anedda et al., 2008). Similar results were obtained by Park et al., where increased ROS synthesis was also observed following metformin exposure using H4II rat hepatocellular carcinoma cells (Park, 2019). Treatment with metformin (dose: 5 mM) stimulated glucose consumption and lactate production, indicating an acceleration of glycolysis. Unexpectedly, metformin markedly increased ROS production. Inhibition of glycolysis by 2-deoxyglucose enhanced metformin’s pro-apoptotic effect, while the antioxidant N-acetylcysteine (NAC) suppressed all pro-apoptotic changes and ROS generation induced by metformin. These findings suggest that metformin promotes apoptosis not by interfering with glycolysis but by enhancing oxidative stress. All studies were summarized in (Table 1).

**TABLE 1:** *In Vitro* studies evaluating the antioxidant action of metformin.

Model	Dose	Parameters	Results	References	Limitations
human umbilical vein endothelial cells	2 mmol/L	increased production of nitric oxide	activated guanosine 5′-triphosphate cyclohydrolase 1	An et al. (2016)	Alteration in eNOS Activity (alteration in the activity of eNOS in response to FG)
		decreased reactive oxygen species production	recoupling of endothelial nitric oxide synthase		Despite the upregulation of phosphorylated eNOS, there are simultaneous changes in the generation of ROS and the production of nitric oxide (NO) under the influence of FG, eNOS becomes uncoupled. Instead of performing its normal role as an enzyme producing NO, eNOS is converted into a molecule that generates superoxide, which is a type of ROS
			inhibited nicotinamide adenine dinucleotide phosphate via an AMP-activated protein kinase		
human aortic endothelial cells	250 µM	reducing reactive oxygen species production, increase in the concentration of the antioxidant thioredoxin	upregulate the expression of the forkhead transcription factor 3 through the AMPK pathway	Hou et al. (2010)	various types of FOXOs may have different, and even
					opposite, effects on ROS level
human endothelial cells	1 mM	reducing reactive oxygen species production	downregulation of NADPH Oxidase 4 activity	Cheng and Lanza-Jacoby (2015)	NA
aortic endothelial cells	1–5 μmol/L	reducing reactive oxygen species production	regulation of NADPH oxidase activity	Ouslimani et al. (2005)	Lack in chelation of intracellular metal ions by Metformin assessment; a contact of cells with culture medium free of metformin for 1 h or 2 h before the experiments
peripheral blood mononuclear human cells	2 µM	Increased of superoxide dismutase, catalase, and glutathione peroxidase activity, reducing reactive oxygen species production	decrease in the expression of NADPH oxidase upregulating key antioxidative enzymes	Bułdak et al. (2016)	Metformin exerts a mild effect on catalase expression, statistically small sample size, a predominant effect of metformin on the catalytic activity of the catalase enzyme
rat adipocytes	4 mM	increased UCP2 levels	UCP adipocytes pathway	Anedda et al. (2008)	The upregulation of UCP2 induced by leptin, when lipolysis and β-oxidation are also promoted, could indicate that there are all involved in energy dissipation (misleading results); polymorphisms in the human UCP2
		increase of ROS			
		decrease in aconitase activity			
H4II rat hepatocellular carcinoma cells	5 mM	Increase in ROS	Apoptosis induction in cancer cells	Park (2019)	unclear by which pathway metformin
		Decrease in NAC			induces ROS generation

FG, fluctuating glucose; NA, not applicable; NAC, N-acetylcysteine; NADPH, nicotinamide adenine dinucleotide phosphate; UCP2, uncoupling proteins; ROS, reactive oxygen species.

Since *in vivo* and *in vitro* studies are inconsistent, broader and more detailed studies focused on the mechanisms responsible for oxidative stress modulation under metformin intervention are still needed (Liu et al., 2021).

### 4.2 *In vivo* studies

In a study by Javadipour et al., a rat model of arsenic-induced diabetes was treated with metformin (dose: 300 mg/kg). The results showed that metformin treatment led to a reduction in oxidative stress in rat pancreatic mitochondria. This effect was achieved through the activation of the SIRT3-related metabolic pathway. SIRT3 is a mitochondrial protein known for its role in regulating cellular metabolism and maintaining mitochondrial function. Activation of the SIRT3 pathway by metformin contributed to the reduction of oxidative stress in the pancreatic mitochondria of the rats with arsenic-induced diabetes. These findings highlight the potential antioxidative effects of metformin via the SIRT3-related metabolic pathway in the context of diabetes induced by arsenic exposure. Further research in this area may provide valuable insights into the therapeutic implications of metformin for managing oxidative stress-related conditions associated with diabetes (Javadipour et al., 2019). In a study conducted by Roxo et al., using a diabetic mouse model, metformin intervention (dose: 250 mg/kg) resulted in notable improvements in lipid metabolism and a reduction in oxidative stress. The beneficial effects were achieved through the restoration of paraoxonase activity. Paraoxonase is an enzyme known for its antioxidant properties and plays a crucial role in protecting cells from oxidative damage. The study demonstrated that metformin treatment enhanced paraoxonase activity, thereby ameliorating oxidative stress in the diabetic mice. The findings from this study provide valuable insights into the mechanisms by which metformin exerts its antioxidative effects and its potential benefits in managing lipid metabolism and oxidative stress in diabetes (Roxo et al., 2019). Moreover, in a study conducted by Lai et al. using a rat model, metformin intervention (dose: 1 mmol/L) was found to be linked to a reduction in oxidative stress. This effect was achieved through the activation of the SIRT3 and AMPK pathways. These findings provide important insights into the antioxidative mechanisms of metformin through the involvement of SIRT3 and AMPK pathways, supporting its potential therapeutic benefits in managing oxidative stress-related conditions (Lai et al., 2016). In the following study Dare et al. investigated the protective effects of ergothioneine (L-egt), a recently FDA-approved bioactive compound, alone or in combination with metformin, on renal damage in a T2DM rat model. The administration of L-egt, either alone or in combination with metformin (dose: 500 mg/kg), reduced hyperglycemia and improved therapeutic outcomes via activation of nuclear factor E2-related factor 2 pathway. Thus, the treatment significantly increased the expression of major antioxidant transcription factors and cytoprotective genes, while decreasing the expression of inflammatory genes in the kidney. The combination of L-egt and metformin related to downregulation of NF-kB/TGF-β1 mRNA expression may enhance therapeutic efficacy and serve as an adjuvant therapy to alleviate renal damage in T2DM (Dare et al., 2021). All studies were summarized in Table 2 (Table 2).

**TABLE 2:** The metformin antioxidant properties based on animal model studies.

Model	Dose	Parameters	Results	References	Limitations
rat model of arsenic-induced diabetes	300 mg/kg	reduction in oxidative stress	SIRT3 pathway activation	Javadipour et al. (2019)	SIRT3 expression elevated parallel to other antioxidant defense component
diabetic mouse model	250 mg/kg	improvements in lipid metabolism and a reduction in oxidative stress	Increased paraoxonase activity	Roxo et al. (2019)	Uncreated metabolic memory associated with diabetes
Rat model	1 mmol/L	Reduction in oxidative stress	activation of the SIRT3 and AMPK pathways	Lai et al. (2016)	the lack of beneficial effects of nitrite and metformin in the more severely affected and older rats
Diabetic rat model	500 mg/kg metformin and L-ergothioneine	significantly increased the expression of major antioxidant transcription factors and cytoprotective genes	Activation of Nrf2 antioxidant	Dare et al. (2021)	experimental phase, no evidence for only metformin beneficial properties, combined therapy may leads to cardiac injury

AMPK, 5′AMP-activated protein kinase; Nrf2, Nuclear factor E2-related factor 2; SIRT3, sirtuin 3.

## 5 Metformin as an antioxidant in clinical trials: advancement in novel approaches

To date, 45 clinical trials evaluating metformin’s antioxidant properties have been registered, with 13 already completed. The most frequently evaluated population in these trials are patients with T2DM. Results from randomized clinical trials have shown that daily intake of 1000 mg of metformin among 50 patients, for 3 months can alleviate oxidative stress and restore antioxidant capacity in newly diagnosed T2DM patients (Mirmiranpour et al., 2013). Similar outcomes were obtained in the study by Pavlovic et al., where metformin intervention resulted in a reduction in erythrocyte and plasma malondialdehyde (MDA) levels and an increase in the erythrocyte activities of Cu, Zn, SOD, CAT, and glutathione levels among newly diagnosed T2DM patients (Pavlovic et al., 2000). These studies' assumptions were expanded in the study conducted by Esteghamati et al., where 99 newly diagnosed T2DM patients were divided into two groups - one undergoing metformin intervention (1,000 mg daily for 12 weeks) and the other undergoing only lifestyle modification. This study revealed that metformin intervention is associated with a greater reduction in the concentrations of advanced oxidation protein products and advanced glycation end products than lifestyle modification alone (Esteghamati et al., 2013). In the following study performed by Chakraborty et al., the effect of metformin on various stress and inflammatory-related parameters in diabetic subjects was evaluated. During the study, 208 T2DM patients were randomly assigned to the metformin and placebo groups, where metformin was supplemented at a daily dose of 2000 mg. The results showed that metformin intervention is associated with a decrease in body mass index (BMI), glycated haemoglobin (HbA1C), low density lipoprotein (LDL) levels, as well as with the reduction in the concentration of oxidative modified advanced protein products and ROS generation (Chakraborty et al., 2011). In the study conducted by Schauer et al., the effect of metformin on vascular and mitochondrial activity was evaluated in patients with type 1 diabetes, considering the origin of increased oxidative stress status. During 6 weeks of metformin intervention (dose: 1,000 mg/daily), mitochondrial complex I, II, III, V, high-sensitive C-reactive protein (hsCRP), glucagon, glycerol and vascular markers, such as endothelin-1 were measured. The study revealed that metformin intervention was associated with an increase in mitochondrial complex protein concentrations, glucagon, glycerol, and endothelin-1 levels, and a decrease in hsCRP concentration. Based on the results obtained, it can be concluded that metformin intervention has a modulatory effect on the oxidative status, but it is not characterized by direct cardiovascular prevention (Abolarin and Schauer, 2024). This means that although metformin influenced various factors related to oxidative stress and vascular activity, its use did not show a direct cardiovascular preventive effect in the context of type 1 diabetes in this particular study.

On the contrary, the study conducted by Diaz-Moralez et al. did not find evidence of metformin’s antioxidant properties. The research involved 72 patients with T2DM, out of which 41 received metformin at a dose of 1700 mg per day for at least 12 months, while 40 age- and sex-matched individuals served as control subjects. Throughout the study, ROS concentration, as well as SOD1 and CAT activities, were monitored for a year, but no significant changes were observed. The study’s results suggest that, in this specific cohort of T2DM patients, metformin did not lead to a notable reduction in ROS concentration or alterations in the activities of SOD1 and CAT, which are key antioxidant enzymes. Thus, it is essential to consider that various factors, including patient characteristics, disease progression, and treatment duration, might influence the antioxidative effects of metformin (Diaz-Morales et al., 2017).

The main ongoing clinical trials based on metformin intervention evaluating antioxidant properties were collected in Table 3 (Table 3).

**TABLE 3:** Ongoing metformin based clinical trials evaluating antioxidant metformin properties.

Title	Number	Dose	Outcome measure	Measure description	Time frame
Independent and Additive Effects Of Micronutrients With Metformin In Patients With PCOS	NCT05653895	1000 mg daily	Insulin Resistance	PCOS associated insulin resistance and hyperinsulinemia by HOMA-IR	6 months
			Menstural Irregularities	Oligo/Anovulation, Questionnaire based history taking of each participant	
			Anti Mullerian Hormone	Measurement of Anti mullerian hormone by ELISA	
			Dehydroepiandrosterone	Measurement of Dehydroepiandrosterone using ELISA	
			Antioxidant Assays perceived stress response (PSS-14) questionnaire	Measurement of Total AntiOxidant Capacity by FRAP ASSAY	
				Measurement MDA by TBARS	
Drug Repurposing Using Metformin for Improving the Therapeutic Outcome in Multiple Sclerosis Patients	NCT05298670	1,000 mg twice daily	Change in IL17 in both arms as measured by ELISA.	Anti-inflammatory marker	After 6 months
			Percentage of Quality of Life deterioration in both arms measured by MSQOL-54	Assessment of quality of life for patients, The highest and lowest values refer to the satisfaction degree of patients	
			Change in IL22 in both arms as measured by ELISA.	Anti-inflammatory marker	
			Malondialdehyde in both arms as measured by Colorimetric tests	Antioxidative marker	
Efficacy of Injectable Vitamin D Supplementation in Females With Polycystic Ovary Syndrome	NCT06045351	750 mg once at dinner for 15 days then twice daily	Hyperandrogenism insulin Resistance	hyperandrogenism by Free Androgen Index via Total Testosterone, Steroid Hormone Binding Globulin	24 weeks
			oxidative stress	HOMA-IR (serum Insulin, Fasting Blood Glucose)	
				Total Antioxidant Capacity	
Therapeutic Intervention of Eriomin Associated With Metformin in the Control of Hyperglycemia in Pre-Diabetic Patients (Eriomin + Met)	NCT06005142	1000 mg daily	Fasting Glycemia	Dosages of glycemia concentration (mg/dL) before and after intervention with Eriomin/placebo	
			OGTT Glycemia	Changes in blood glucose 2 h after the OGTT (mg/dL) before and after intervention with Eriomin/placebo	
			HBA1C	Dosages of HBA1C (%) in the blood serum/plasma before and after intervention with Eriomin/placebo	
			Insulin	Dosages of Insulin (µU/mL) in the blood serum/plasma before and after intervention with Eriomin/placebo	
			Blood Lipids profile		
			Antioxidant Capacity	Dosages of cholesterol (mg/dL), HDL-cholesterol (mg/dL) and triglycerides (mg/dL) in the blood serum/plasma before and after intervention with Eriomin/placebo	
			Lipid Peroxidation	Dosages of TEAC (μM) in the blood serum/plasma before and after intervention with Eriomin/placebo	
				Dosages of MDA (mM) the blood serum/plasma before and after intervention with Eriomin/placebo	

ELISA, Enzyme-Linked Immunosorbent Assay; FRAP ASSAY, ferric reducing ability of plasma assay; HBA1C, glycated hemoglobin; HDL, High-Density Lipoprotein; HOMA-IR, homeostatic model assessment for insulin resistance; IL17, Interleukin 17; IL22, Interleukin MDA, malondialdehyde; mg, milligram; MSQOL-54, Multiple Sclerosis Quality of Life-54; NCT, ClinicalTrials.gov Identifier; OGTT, oral glucose tolerance test; PCOS, polycystic ovary syndrome; TEAC, trolox equivalent antioxidant capacity; TBARS, thiobarbituric acid reactive substances.

## 6 The future of antioxidant treatment discussion: considerations and opportunities for metformin intervention

Since oxidative stress is involved in the pathogenesis of many diseases, it is crucial to find ways to overcome its negative impact (Blacker and Duchen, 2016; Buczyńska et al., 2021a; Adamska et al., 2021; Buczyńska et al., 2021b; Buczyńska et al., 2023a; Buczyńska et al., 2023b; Buczyńska et al., 2023c; Kościuszko et al., 2023). This review focused on presenting new and promising mechanisms responsible for the potential antioxidant use of metformin, while forecasting further research directions (Salvatore et al., 2021). Despite the promising results obtained during *in vivo* research, several studies have revealed inconsistencies regarding the antioxidant effect of metformin. The study conducted by Sakellakis et al. underscored that metformin exhibits a characteristic tendency to modulate oxidative phosphorylation (OXPHOS) with the compound’s clinical relevance being widely recognized. Nonetheless, the therapeutic efficacy of metformin in OXPHOS inhibition for cancer patients remains enigmatic. The existing body of evidence indicates that metformin, when administered at therapeutic doses, maintains its plasma concentration within the micromolar range. Although isolated mitochondria necessitate millimolar concentrations for the inhibition of Complex I activity, there exists no substantiated proof supporting the mitochondrial accumulation of metformin. Metformin exerts a subtle impact on the adenosine diphosphate to adenosine triphosphate ratio, culminating in the activation of AMP-activated protein kinase, thereby fostering the activation of ATP-generating catabolic pathways and restoration of cellular energy homeostasis. The metformin’s value as an OXPHOS inhibitor for cancer treatment remains a subject of contention, and prudence is advisable in its utilization for this purpose (Sakellakis, 2023). Thus, molecular alterations under metformin intervention have not clearly demonstrated antioxidant properties. Although multiple reports provide evidence that oxidative stress markers unequivocally decrease in metformin-treated patients, the molecular targets have not been comprehensively determined. Nonetheless, several studies have provided evidence supporting metformin’s involvement in reducing oxidative stress in various cellular contexts. Metformin intervention has been shown to increase the concentrations of mitochondrial complex proteins and glucagon, along with glycerol levels. Conversely, it leads to a decrease in the generation of ROS and levels of hsCRP, and advanced oxidation protein products (AOPP). These findings collectively suggest that metformin has antioxidative effects that extend beyond the modulation of ROS generation. Metformin’s ability to enhance mitochondrial complex proteins and influence various biomarkers related to oxidative stress reflects its potential in managing oxidative status in different physiological conditions. However, further research is needed to comprehensively understand the underlying mechanisms and the extent of metformin’s antioxidative impact in various disease states and patient populations (Abolarin and Schauer, 2024). Hence, metformin is a potential target that protects cells from mitochondrial injury, increases oxidative stress, and attenuates inflammation (Ghavimi et al., 2018). Given the fact that metformin is characterized by pleiotropic properties, including the ability to increase antioxidant ability, it is a promising candidate for preventing many diseases.

Since metformin intervention is safe and well-tolerated among patients, determining the most effective dose in terms of beneficial effects and disease prevention is a significant challenge (Chang et al., 2018). However, determining clinically beneficial doses of metformin is challenging due to the unknown portal vein and liver concentrations of metformin following oral ingestion in humans (Lamoia and Shulman, 2021). Furthermore, since metformin is excreted by the kidneys, patients with eGFR <30 mL/min/1.73 m^2^ should not receive metformin due to the risk of metformin-associated lactic acidosis (MALA) (Bakris and Molitch, 2016). Additionally, metformin intervention has been associated with vitamin B12 deficiency, so patients at particular risk for this vitamin deficiency should be supplemented with multivitamins to protect against deficiency (Kim et al., 2019).

## 7 Limitation of metformin use

Metformin, as a popular drug used in the treatment of T2DM, has its application and benefits in improving glycemic control and metabolism in many patients. However, there are certain patient groups and circumstances in which metformin may be inappropriate or require special caution in use. The first group of patients for whom metformin may be inappropriate is individuals with specific kidney conditions (Chen et al., 2013). Since metformin is primarily eliminated through the kidneys, patients with kidney impairment, especially in advanced stages, may be more susceptible to increased drug accumulation and potential adverse effects. In such cases, adjusting the metformin dose or avoiding its use altogether is necessary. Additionally, patients with advanced liver conditions should also avoid metformin or exercise extra caution. The liver plays a significant role in metformin metabolism, and liver dysfunction may lead to drug accumulation and potential side effects. In cases of advance cardiovascular diseases and heart failure, caution is also required in metformin use. Some clinical studies suggest that metformin may have both favorable and unfavorable effects on the cardiovascular system (Graham et al., 2011; Polverino et al., 2021; Sakouhi et al., 2023). Despite these limitations, metformin remains a safe and effective drug for a large number of patients with T2DM, where clinical studies confirm its benefits in various diseases.

## 8 Conclusion

The investigation of metformin as an antioxidant intervention has yielded promising outcomes in various studies both *in vitro* and *in vivo*. Metformin demonstrates antioxidative effects through diverse molecular pathways, including SIRT3 and AMPK pathway activation, restoration of paraoxonase activity, and downregulation of NADPH oxidase expression. These mechanisms collectively contribute to a decrease in reactive oxygen species production and an enhancement of oxidative stress status across different cellular contexts and disease models. However, it is crucial to acknowledge the variability in reported results. While some studies highlight metformin’s antioxidative properties, others indicate increased ROS production or decreased antioxidative enzyme activities. Such discrepancies may stem from differences in study design, patient characteristics, and specific oxidative stress context. Moreover, the effectiveness of metformin as an antioxidant intervention is influenced by factors such as dosage, treatment duration, and underlying medical conditions. Patients with T2DM, metabolic syndrome, or certain kidney or liver impairments may exhibit varied responses to metformin’s antioxidative effects. Despite these complexities, metformin remains a widely used and well-tolerated drug for managing T2DM, with potential benefits extending beyond glycemic control. Its role as an antioxidant intervention, particularly in diseases like cancer prevention and aging-related conditions, is actively researched. To comprehensively understand and harness metformin’s antioxidative potential, additional multicenter clinical trials involving diverse patient populations and varied medical contexts are essential. Long-term studies investigating its impact on cardiovascular health, cancer risk reduction, and other oxidative stress-related conditions will significantly contribute to determining its clinical utility as an antioxidant intervention.

## Data Availability

The original contributions presented in the study are included in the article/Supplementary material, further inquiries can be directed to the corresponding authors.
